# Supplementary material for: Modulation of prion protein expression through cryptic splice site manipulation
Source: bioRxiv. 2023 Dec 19:2023.12.19.572439. Preprint. [Version 1] doi: 10.1101/2023.12.19.572439 (PMC10769280; doi:10.1101/2023.12.19.572439)
Supplement: Supplement 2 [file NIHPP2023.12.19.572439v1-supplement-2.pdf]

bioRxiv preprint doi: https://doi.org/10.1101/2023.12.19.572439; this version posted December 19, 2023. The copyright holder for this preprint (which was not certified by peer review) is the author/funder, who has granted bioRxiv a license to display the preprint in perpetuity. It is made available under aCC-BY 4.0 International license.

SUPPLEMENT

Supplementary tables 1-6 provided as a separate Excel file.

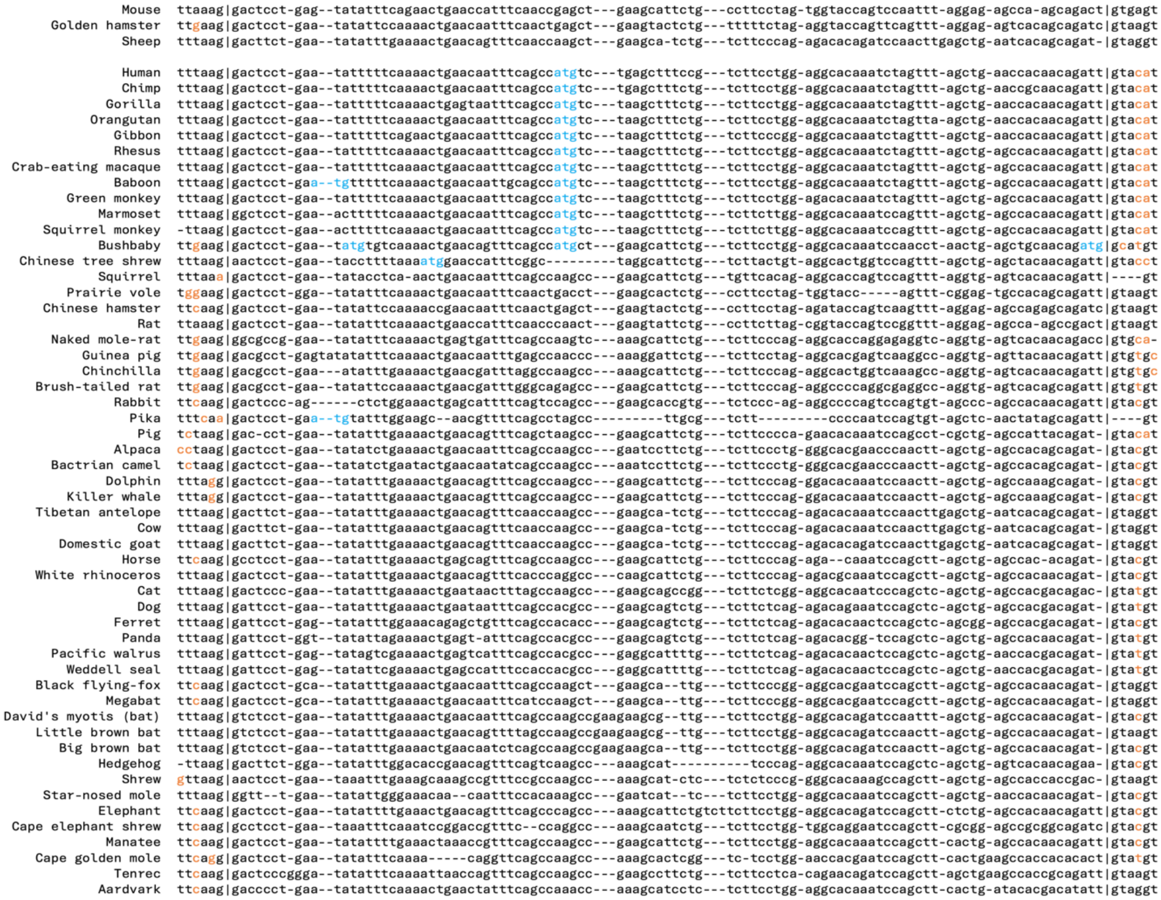

Figure S1. Multiple alignment of PRNP exon 2 orthologous sequence for all available eutherian mammals. *As in Figure 1D, but including eutherian mammals without ATGs in exon 2. Lesser Egyptian jerboa is excluded because orthologous sequence was identified for only part of exon 2.*

bioRxiv preprint doi: https://doi.org/10.1101/2023.12.19.572439; this version posted December 19, 2023. The copyright holder for this preprint (which was not certified by peer review) is the author/funder, who has granted bioRxiv a license to display the preprint in perpetuity. It is made available under aCC-BY 4.0 International license.

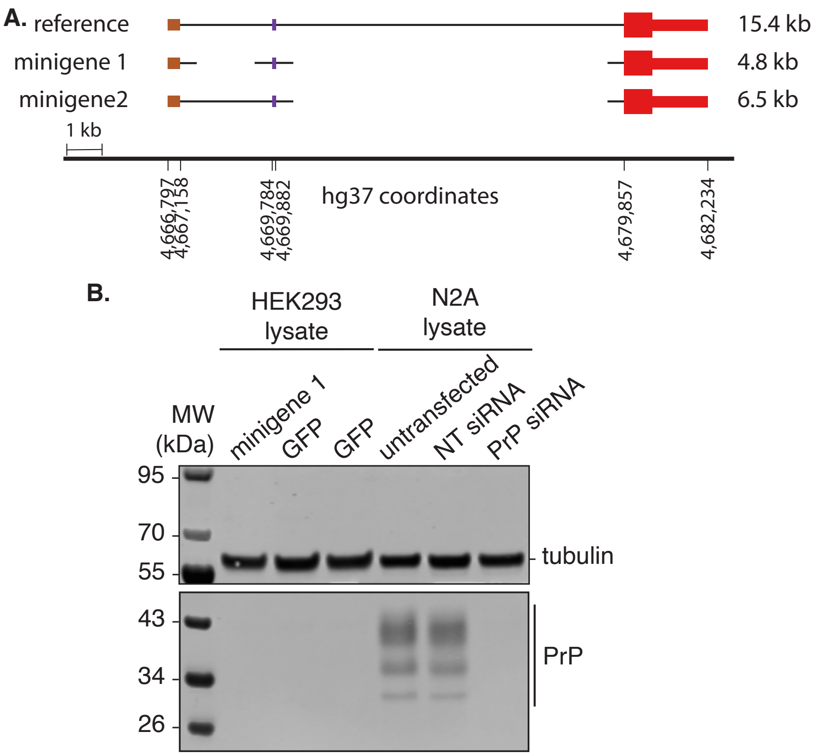

Figure S2. Alternative minigene construct tested in cells. A) *Comparison of human reference sequence with an alternative "minigene 1" containing only 500 bp at either end of intron 1, and the "minigene 2" used throughout the main text of this manuscript.* ***B)*** *Immunoblot failing to detect any expression of minigene 1 in transfected HEK293 cells. Primary antibody: 6D11, see Methods.*
